# Quantitative DCE-MRI: an efficient diagnostic technique for evaluating early micro-environment permeability changes in ankylosing spondylitis

**DOI:** 10.1186/s12891-020-03805-1

**Published:** 2020-11-24

**Authors:** Hui Yang, Ling Jiang, Jiang Li, Xiuzhu Zheng, Qianqian Yao, Changqin Li, Jianzhong Zhu, Jian Qin

**Affiliations:** 1 Department of Radiology, The Second Affiliated Hospital of Shandong First Medical University, Tai’an, 271000 Shandong China; 2 Department of Medical Equipment, The Second Affiliated Hospital of Shandong First Medical University, Tai’an, 271000 Shandong China

**Keywords:** Ankylosing spondylitis, Sacroiliac joint, Dynamic contrast-enhanced magnetic resonance imaging, Quantitatively micro-environment, Animal model

## Abstract

**Background:**

In the management of early inflammatory joint of ankylosing spondylitis (AS), there is a need for reliable noninvasive quantitative monitoring biomarker to closely assess status of synovitis progression. Cognizant to this,studies geared on improving techniques for quantitative evaluation of micro-environment permeability of the joint space are necessary. Such improved techniques may provide tissue perfusion as important biological parameters and can further help in understanding the origin of early changes associated with AS. The purpose of this study was to prospectively evaluate the diagnostic performance and determine longitudinal relationships of early micro-environment active in the joint space of the sacroiliac joint (SIJ) with a rat model by using quantitative dynamic contrast-enhanced magnetic resonance imaging (DCE-MRI).

**Methods:**

Thirty wistar male rats were randomly assigned to the model (*n* = 15) or control (*n* = 15) group. All rats underwent DCE-MRI of SIJ region at fixed time points (12, 17 and 22 weeks),between September 2018 and October 2019. Differences in permeability parameters between the two groups at the same time point were compared by using an independent samples *t* test. Spearman correlations of DCE-MRI parameters with different time points in model group were analyzed. All statistical analyses were performed with software.

**Results:**

At 12 weeks,the K^trans^,K_ep_ and V_e_ values in the model group were slightly lower than those in control group,but all the differences were not statistically significant (*p* > 0.05). Compared with control group,the transfer constant (K^trans^) values increased significantly at 17 weeks and 22 weeks in model group,while the rate constant (K_ep_) and volume of extravascular extracellular space (V_e_) significantly increased only at 22 weeks(*p* < 0.05). The K^trans^,K_ep_ and V_e_ were positively correlated with increasing time points (*r* = 0.946, *P*<0.01 for K^trans^; *r* = 0.945, *P*<0.01 for K_ep_; and *r* = 0.832, *P*<0.01 for V_e_).

**Conclusion:**

Quantitative DCE-MRI parameters are valuable for evaluating the early longitudinal relationship of micro-environment permeability changes in the joint space of SIJ.

## Background

Ankylosing spondylitis (AS) is a chronic inflammatory disease that affect approximately 0.1–2% of the general population [1]. It mostly affects the young adults and its etiology is still unknown. Early detecting and monitoring the progress of abnormal changes associated with AS are paramount importance because any delays in accurate diagnosis can result in serious disabilities and economic cost [2]. Cognizant to this,there is still the need to search for methods that improve the diagnostic accuracy for recognition of changes in the early phase of AS which can help in identifying patients significantly benefit from timely clinical interventions.

Sacroiliitis is the most important clinical manifestation of AS [3].However,there is high variability in detecting the activity of sacroiliitis by clinical methods such as C-reactive protein (CRP) and erythrocyte sedimentation rate (ESR) [4]. Evaluating active progression of AS by magnetic resonance imaging (MRI) had been widely accepted,which is a sensitive, non-invasive method that can permit the visualization of active and structural lesions,even in the absence of radiographically evident disease [5–10]. However,the MRI assessment relies on visual comparison of differences in image intensity to assess inflammation changes of the SIJ still remained subjective,limited by the inability to provide objective quantitative parameter. More recently,the Spondyloarthritis Research Consortium of Canada (SPARCC) SIJ Structural Score (SSS) is usually used to evaluate the sacroiliac joints [11]. What is noteworthy is that this system is also subjective and influenced by the radiologist’s experience,especially more challenging in a part of people with less structural damage and that recognition of structural lesions [12–14].

Compared with morphological imaging,quantitative dynamic contrast enhanced magnetic resonance imaging (DCE-MRI),basing on a pharmacokinetic model,have been successfully used to assess changes in different diseases [3, 15, 16]. It can provide sufficient information on microenvironment changes and could be used to assess the severity of inflammation quantitatively [17]. In the management of early AS joint, there is also a need for reliable noninvasive quantitative monitoring biomarker to closely assess status of inflammatory progression. In 2019, the ASAS-MRI work group have pointed out that increased signal on contrast-enhanced images of the joint space of the cartilaginous portion of the SIJ reflected inflammation at the osteochondral interface,as this would be consistent with the understanding of early histopathological feature of AS [18]. Cognizant to this,studies geared on improving techniques for quantitative evaluation of micro-environment permeability of the joint space before edema and inflammation visualized in adjacent bone marrow of SIJ are necessary. Such improved techniques may provide tissue perfusion as important biological parameters and can further help in understanding the origin of early changes associated with AS.

Because it is difficult to obtain human tissue samples of the SIJ, the understanding of early AS pathogenesis greatly depend on animal models. The purpose of this study was to utilize quantitative DCE-MRI to dig deeper into investigating the longitudinal relationship of early micro-environment permeability in the joint space of SIJ at fixed time points (12, 17 and 22 weeks) before onset of active bone marrow changes in an AS rat model.

## Methods

### Animals

Our study began in September 2018 and ended in October 2019.All experiments were approved by the Institutional Animal Experiment Center and were performed in accordance with the Guide for the Care and Use of Laboratory Animals [19]. Thirty six-week-old male wistar rats (Weight,160-200 g;Department of Experimental Animal breeding Co., Ltd) were used in the study. Each cage housed five rats at 20 °C to 25 °C, with a 12 h light-dark cycle and standard laboratory rat diet and water were available ad libitum.

### Rat AS model

The experiment was performed when all rats had been acclimatized in a Specific Pathogen Free (SPF) environment for 1 week. Thirty male wistar rats were randomly divided into 2 groups, including the model group(*n* = 15) and control (*n* = 15) group. The AS models were established by reference to the past successful experience [10],as follows:in the model group,1 mg proteoglycan protein was dissolved in 1 ml sterile normal saline with 1 ml Freund’s complete adjuvant (FCA) and was immediately administered intraperitoneally with 0.2 ml for each rat,then followed with mixed solvent consisted of the same volume of proteoglycan protein and Freund’s incomplete adjuvant (FIA) after 2 weeks and 4 weeks respectively. The control group were injected the same amount of saline instead of proteoglycan by the same way and time point. Each rat was weighted before the MRI scan. At 12,17, 22 weeks after the last induction,five rats at each time point of each group were randomly selected for MRI examination (from 5 p.m. to 10 p.m) under anesthesia (3 ml of Urethane intraperitoneal injection, Shanghai Shanpu Chemical Co., Ltd), then euthanized and sent for pathology acquisition.

### MR examination

MRI examination was performed on a 3.0-T imager (GE Discovery 750; GE Healthcare) with a matched eight-channel animal coil (Wankang Medical Technology Co., Ltd., China) of the bilateral SIJ at 12,17, 22 weeks after the last induction. MRI sequences were obtained using the following protocols:①Axial T2-weighted with fat-saturated [repetition time/echo time (TR/TE):3000/96 ms;matrix size:192 × 192;slice thickness:0.8 mm;field of view (FOV):6.0 × 6.0;number of excitations (NEX):4];②Coronal fs T1 FSE (TR/TE:500/13.5 ms; matrix size:192 × 192;slice thickness:0.8 mm;FOV:6.0 × 6.0;NEX:4);③DCE-MRI was performed using 3D liver acquisition with volume acceleration-flexible (LAVA-Flex),which consisted of a pre-contrast T1 mapping sequence and a dynamic sequence,and the parameters as follows: TR/TE: 5.7/2.0 ms; matrix size: 128 × 128;slice thickness:2.0 mm;FOV:12 × 12; NEX:2. Prior to contrast administration,the T1 mapping was performed with multiple flip angles. Dynamic sequence was performed with the same parameters as T1 mapping but with flip angle 12°.When the scan for the eighth phase was started,1 ml gadopentetate dimeglumine contrast agent (BeiLu Pharmaceutical Co.,Ltd.,Beijing,China; dose:0.5 mmol/kg) was rapidly injected manually,followed by 2 ml saline flush into the tail vein through a intravenous catheter. The temporal resolution was 4 s and the total scan time was 5 min 20 s including 80 phases.

### Image analysis

The quantitative DCE-MRI analysis was processed on the Advantage Workstation (ADW4.7version,GE,US). The DCE-MRI pharmacokinetics parameters were computed by using two-compartment Standard Toft’s Model [20]. A manual determination of the arterial input function (AIF) in rat MRI is often difficult because of the small spatial dimensions,so the AIF were automatically calculated by Model-based Mode in the Advantage Workstation and determined from the following equations:

$$ {\displaystyle \begin{array}{l}\mathrm{d}\;{\mathrm{C}}_{\mathrm{t}/\mathrm{dt}}={\mathrm{K}}^{\mathrm{t}\mathrm{rans}}{\mathrm{C}}_{\mathrm{p}}\hbox{-} {\mathrm{K}}_{\mathrm{e}\mathrm{p}}{\mathrm{C}}_{\mathrm{t}};\\ {}{\mathrm{C}}_{\mathrm{t}}\left(\mathrm{t}\right)={\mathrm{K}}^{\mathrm{t}\mathrm{rans}}\left[{\mathrm{C}}_{\mathrm{p}}\left(\mathrm{t}\right)\otimes {{{\mathrm{e}}^{\hbox{-} \Big(\mathrm{K}}}_{\mathrm{e}\mathrm{p}}}^{\mathrm{t}\Big)}\right];\\ {}{\mathrm{K}}^{\mathrm{t}\mathrm{rans}}={\mathrm{V}}_{\mathrm{e}}\times {\mathrm{K}}_{\mathrm{e}\mathrm{p}};\end{array}} $$

where dC_t/dt_ is the integration of concentration with time, K^trans^ as the volume transfer constant of the contrast agent from Vascular space (VS) to Extra-vascular extra-cellular space (EES),K_ep_ as the rate transfer constant of the contrast agent from EES to VS,C_p_ as the plasma tracer concentration, Ct as the concentration of contrast agent in the tissue, ⓧ as the calculation of convolution, and V_e_ as the volume of EES per unit volume of the contrast agent in the tissue.

**Fig. 1:**
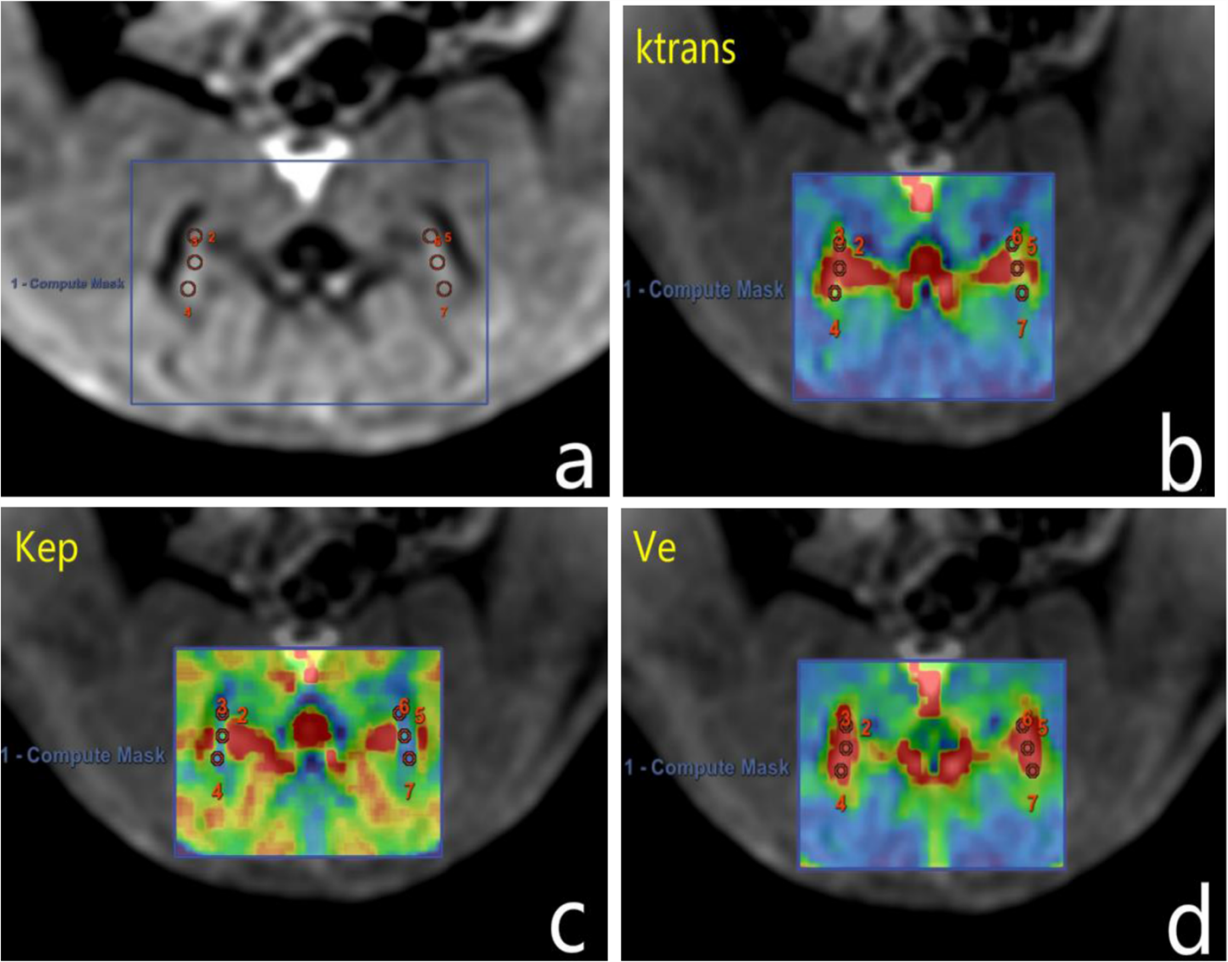
Representative images of ROIs outlined. **a** Image shows that ROIs have been manually drawn on magnified DCE-MRI image,and that the colour parametric maps of (**b**) K^trans^ (min^− 1^), (**c**) K_ep_ (min^− 1^) and (**d**) V_e_ are automatically generated

All MRI examinations were independently processed by two radiologists (with 15 years and 8 years of experience in reading musculoskeletal system MR images with the doubleblind method). Regions of interest (ROI) were manually positioned in the upper,middle and lower third of the joint space with the maximum transverse level of SIJ on magnified DCE-MRI images and then the ROIs were copied and pasted onto the K^trans^ maps, K_ep_ maps, and V_e_ maps (Fig. 1). Total of 3 ROIs (2-4 mm^2^) for each side and then the bilateral average values were calculated for analysis,as shown in Fig. 1.

### Histologic assessment

All rats of each time point were intraperitoneally injected with 1% pentobarbital (Sigma company) to euthanize them. The SIJ were then cut across the midline and removed. They were fixed in 10% formalin for 1 or 2 days and the acid-decalcified with 10% methanoic acid for 1 week. They were then embedded in paraffin and dehydrated in graded ethanol. The tissues were then cut into sections and stained with hematoxylin for 8 min and the with eosin for 2 min. The pathological changes of the SIJ were observed under a microscope (MODEL BX53F, OLYMPUS, Tokyo,Japan) by an experienced pathologist without knowledge of the final group allocation status.

### Statistical analysis

The IBM SPSS 23.0 software (Armonk,NY) and Med Calc 15.8 (Mariakerke,Belgium) were used for statistical analyses. Quantitative parameters are expressed as the means±standard deviation. Data were tested for normality analysis using the Kolmogorov–Smirnov test and then with the Levene test for variance homogeneity analysis. Differences in permeability parameters between the two groups at the same time point were compared by using an independent samples *t* test. In order to evaluate interobserver variability, the coefficient of variation (CV) was calculated for the two sets of measurements. Interobserver agreement was evaluated using interclass coefficient correlation (ICC) and Bland-Altman analysis. Spearman correlations of DCE-MRI parameters with different time points in model group were analyzed. *P* values of less than 0.05 were considered as statistically significant.

## Results

### Interobserver reproducibility

**Fig. 2:**
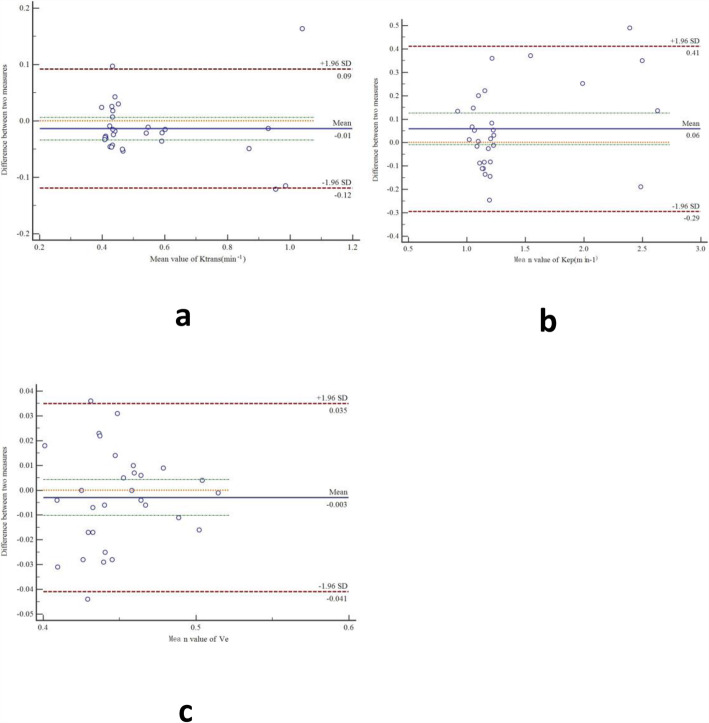
Bland-Altman plots show interobserver reliability for measurement of DCE-MRI parameters (**a**-**c**). SD = standard deviation

Table 1 revealed the measurements of the DCE-MRI parameters (K^trans^, K_ep_ and V_e_) with interclass coefficient correlation ranging from 0.78 to 0.93.The coefficient of variation (CV) ranged from 3.1 to 9.6%.Bland-Altman plots showed good agreement between interobserver DCE-MRI parameter measurements (Fig. 2).

**Table 1:** Interobserver reproducibility in the assessment of DCE-MRI parameters

Parameter	Interclass coefficient correlation (95% CI)	Coefficient of variation (%)
K^trans^ (min ^−1^)	0.92 (0.86–0.95)	7.1
K_ep_ (min ^−1^)	0.93 (0.85–0.96)	9.6
V_e_	0.78 (0.59–0.89)	3.1

Data are presented as means (95%CI) or n(%). Interobserver agreement was evaluated by using interclass coefficient correlation (ICC) analysis and interobserver variability was calculated by using the coefficient of variation (CV)

### DCE-MRI parameters

None of the rats had severe adverse effects in the process of conducting this study. All rats entered into the procedures survived to the end and were actually included in the analyses except for one rat at 17 week,which only unilateral SIJ was analyzed. All imaging examinations were performed successfully at each different time point. There were no positive findings of adjacent bone marrow signal intensity changes in fs axial T2WI and coronal fs T1WI images by visual observation in all the rats of model and control groups. On the DCE-MRI pictures,different degrees of increased signal of the joint space of the cartilaginous portion of the SIJ can be seen.

**Fig. 3:**
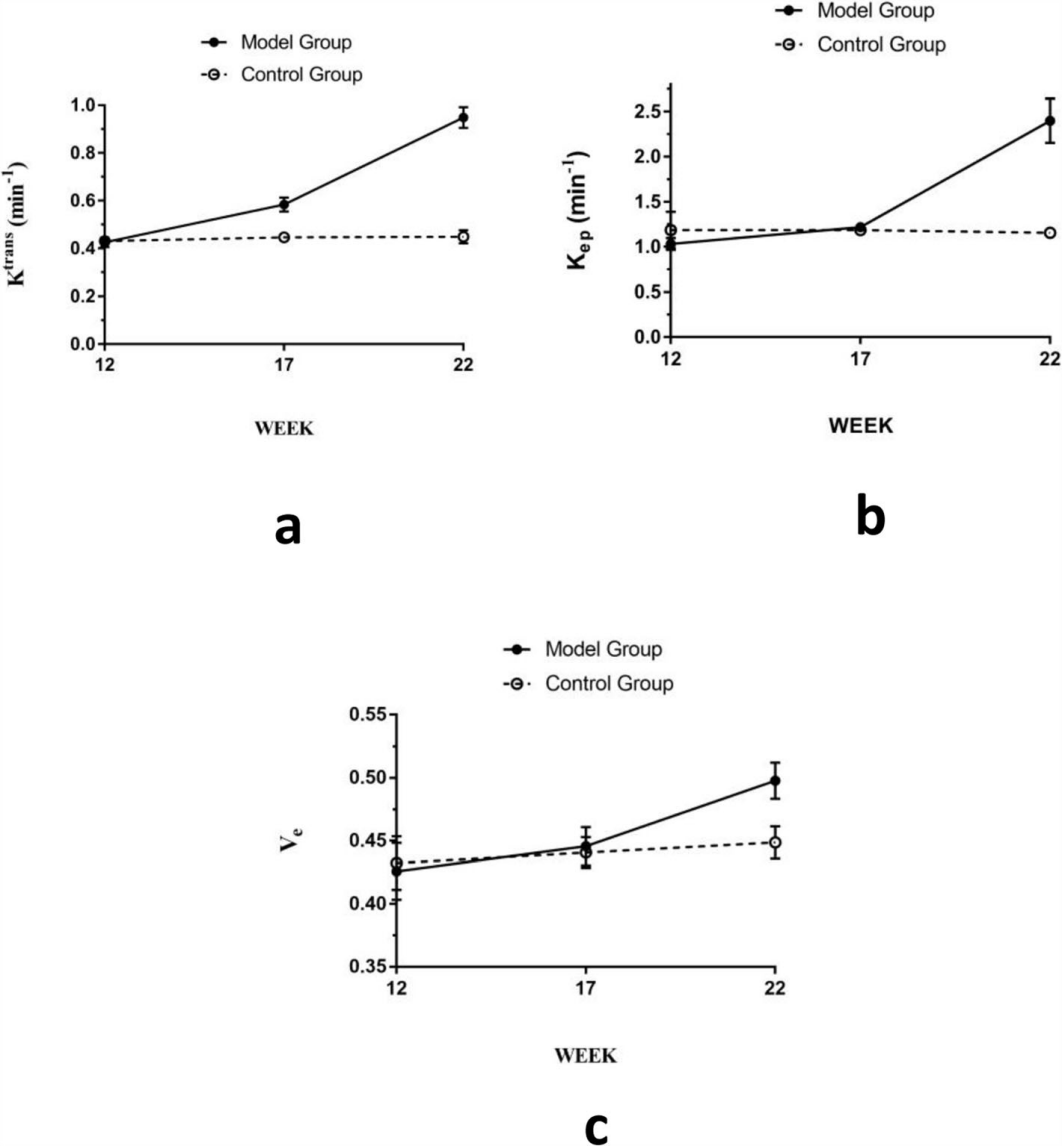
Graphs show longitudinal DCE-MRI parameters in the model and control groups of wistar rats. At 12 weeks,all the differences of K^trans^ (min^− 1^),K_ep_ (min^− 1^) and V_e_ values were not statistically significant between the model and control groups. Compared with control group, **(a)** the K^trans^ (min^− 1^) was significantly increased at 17 weeks and 22 weeks in model group,while**(b)** K_ep_ (min^− 1^) and **(c)** V_e_ were significantly increased only at 22 weeks

The control group had no significant differences in DCE-MRI parameters over time. With the increase of weeks,all the DCE-MRI parameters gradually increased at different degrees of model group (Table 2). At 12 weeks,the K^trans^,K_ep_ and V_e_ values in the model group were slightly lower than those in control group,but all the differences were not statistically significant (*p* > 0.05). Compared with control group, the K^trans^ was markedly and significantly increased at 17 weeks and 22 weeks in model group,while K_ep_ and V_e_ were significantly increased only at 22 weeks(*p* < 0.05) (Table 2) (Fig. 3). To evaluate the DCE-MRI parameters as monitoring progression markers of early micro-environment permeability changes in the joint space of SIJ,we correlated them with different time points on behalf of different stages of inflammation in model group by reference of pathological findings. We found a statistically significant positive correlation between the K^trans^, K_ep_ and V_e_ with increasing time points (*r* = 0.946, *P*<0.01 for K^trans^; *r* = 0.945, *P*<0.01 for K_ep_; and *r* = 0.832, *P*<0.01 for V_e_).

**Table 2:** DCE-MRI parameters in the model and control groups at each time point

Time Point	Model Group	Control Group	*P*	95% Confidence Interval for Difference
K^trans^ (min ^− 1^)				
Week 12	0.42 ± 0.02	0.43 ± 0.02	0.736	− 0.024 ~ 0.033
Week 17	0.58 ± 0.03	0.44 ± 0.01	0.000^*^	−0.174 ~ − 0.100
Week 22	0.94 ± 0.04	0.44 ± 0.03	0.000^*^	−0.552 ~ − 0.446
K_ep_ (min ^−1^)				
Week 12	1.02 ± 0.06	1.18 ± 0.20	0.143	−0.065 ~ 0.376
Week 17	1.21 ± 0.01	1.18 ± 0.03	0.083	−0.071 ~ 0.005
Week 22	2.39 ± 0.24	1.15 ± 0.04	0.000^*^	−1.498 ~ −0.988
V_e_				
Week 12	0.42 ± 0.02	0.43 ± 0.02	0.656	−0.025 ~ 0.038
Week 17	0.44 ± 0.01	0.44 ± 0.02	0.581	−0.025 ~ 0.015
Week 22	0.49 ± 0.01	0.45 ± 0.01	0.000^*^	−0.068 ~ − 0.029

Data are presented as as means±standard deviation. *P* values for differences between different groups of same time point were determined by independent samples *t* test.*Statistically significant differences

### Finding at histopathology

Model group: At week 12,positive histologic evidence of inflammation was entirely absent in synovium,cartilage,subchondral bone and bone marrow of bilateral SIJ in 5 rats. At week 17,inflammatory synovium were present in 4 rats of bilateral SIJ and one rats of unilateral SIJ. At week 22,definite pannus forming of synovium could be seen in 5 rats of bilateral SIJ with 2 rats had hemorrhage and slightly damage on the surface of cartilage.

Control group: There are no positive histologic evidence of inflammation of all rats in each time point.

## Discussion

In our study,the potential of quantitative DCE-MRI analysis to elucidate the longitudinal relationship of early inflammation micro-environment permeability in the joint space of SIJ before onset of active structural changes and bone destruction was well demonstrated.

AS is a chronic progressive rheumatic disorder that affects the SIJ. It is a challenge to recognize the disease early and thus proper diagnosis is often made long after manifestation of symptoms [21]. Inflammation is an important indicator of the activity of AS. It plays a key role in early identification and the earlier detection of synovitis is important to prevent the development of structural changes. MRI has become particularly crucial in early disease detection without the risk of radiation exposure and follow-up SpA sacroiliitis [22–24]. Understanding the pathophysiologic relationship between early inflammation micro-environment changes in the SIJ and the role of MRI in accurate monitoring of disease progression can significantly improve the understanding of disease evolution. Our study was aimed at evaluating early changes of AS by using functional MRI sequences in a rat model and providing a basis for further clinical study.

K^trans^, K_ep_ and V_e_ parameters reflect perfusion and permeability,the reverse transportation of gadolinium chelate back to the vascular space as well as extravascular extracellular space volume. In the present study,there were no significant differences between the model group and control group based on DCE-MRI parameters assessing in the joint space of SIJ at 12 weeks after induction. These results were consistent with the pathological results which showed no significant positive inflammatory changes at 12 weeks. At 17 and 22 weeks,the differences of K^trans^ between the two groups were statistically significant. The previous investigations have noted that in early stages of axial SpA, pannus formation helps in understanding the associated pathological changes [25]. It is characterized by formation of high vascular granulation tissue by the inflamed synovium and/or subchondral bone marrow. Inflammation may subsequently trigger the formation of angiogenesis by increasing the density of the micro-vessels that provide a channel for expression of mediation and invasion of inflammatory cells and this in turn exacerbates local damage to the cartilage [26, 27]. Based on pathological results,local infiltration of inflammatory tissues of the subsynoviocytic and areolar connective tissue in the early stage may led to the increase of vascular permeability. A related view is that François R J et al. [28] have reported that a small number of lymphocytes and plasma cells with a large number of macrophages infiltrated the subsynoviocytic and areolar connective tissue in a systematic histological study of early SIJ.

At 22 weeks, except for the K^trans^, K_ep_ and V_e_ also significantly increased in the model group than control group. These changes indicated increased permeability and extravascular extracellular space volume of the capsulitis and synovitis in SIJ. This was attributed to infiltration of the inflammatory cells and pannus formation in the synovial area with increased microcirculation and greater capillary permeability. This result was consistent with that of Zhang et al. [3] who found that K^trans^, K_ep_ and V_e_ of AS patients in the active group were significantly higher than those of AS patients in the inactive group patients using quantitative DCE-MRI.These results revealed that quantitative DCE-MRI parameters could differentiate between active and inactive AS. Higher K^trans^ and K_ep_ values are associated with increased microcirculation and greater capillary permeability of inflammatory tissues in the active sacroiliitis. Another previous study [29] has shown DCE-MRI parameters of the K^trans^,k_ep_, and V_e_ could be used to detect synovial inflammation in patients with early arthritis and correlated with synovial expression of the endothelial cell (EC) marker von Willebrand factor (vWF),which could facilitate the evaluation of joints inaccessible to next proper clinical examination. Our result was similar to the above report that quantitative DCE-MRI can provide performance for detecting early inflammation micro-environment permeability in SIJ of AS. In addition, there was a statistically significant positive correlation between the K^trans^, K_ep_,V_e_ with increasing weeks and changes in the K^trans^ occurred earlier than K_ep_ and V_e_. Based on our results,the K^trans^ was more sensitive than the other parameters and it may has a potentially efficacy to predict early inflammatory activity more timely.

We evaluated the interobserver variability for quantitative DCE-MRI parameter measurements. Our results indicated good agreements between the two radiologists for the measurements of quantitative DCE-MRI parameters. Because the accuracy of the result is highly depend on ROI delineating, in order to minimize the selection bias,we placed the ROIs in the upper,middle and lower third of the joint space with the maximum transverse level of SIJ on contrast-enhanced images by the reference of increased signal of the joint space [18]. However, standardization of strategies for ROI determination should be done in other subsequent in depth studies.

Nevertheless,this study was limited by several factors. Firstly,changes in DCE-MRI parameters were not assessed after the 22nd week. The sample size used was relatively small and thus further prospective analyses of a larger number of samples is needed to validate our results. Secondly,in our previous work [10],we have successfully investigated the diagnostic performance of semi-quantitative DCE-MRI in detecting the early activity of sacroiliitis in AS and this present study is the extension of earlier work. However,the correlation of semi-quantitative and quantitative DCE-MRI parameters were not evaluated. In the future,the performance comparison of different contrast-enhanced models should be investigated. Moreover,the higher magnification MR images were not compared with the corresponding pathological images in the present study and it was our ongoing study in depth. Thirdly,none of the MRI denoising methods were used in our present study. In the next study,the appropriate MRI denoising methods should be given special attention in order to enhance image quality with taking the global and local image features into account. Finally,when applying a model selection technique to DCE data, the duration of dynamic acquisition,temporal resolution,the underlying physiology and the parameters of models all need to be considered [30],so the more appropriate model should be further studied and compared by a large sample.

## Conclusions

In conclusion,quantitative DCE-MRI parameters are valuable for evaluating the early micro-environment permeability changes in the joint space of SIJ. In particular, K^trans^ is a sensitive and timely index for demonstrating early inflammatory activity in AS,which may be an useful method for clinician to take effective measures with suspected synovitis in patients at risk of developing AS.

## Data Availability

The datasets used and/or analyzed during the current study are available from the corresponding author on reasonable request.

## Abbreviations

DCE-MRI: Dynamic contrast-enhanced magnetic resonance imaging
SIJ: Sacroiliac joint
AS: Ankylosing spondylitis
CRP: C-reactive protein
ESR: Erythrocyte sedimentation rate
LAVA: Liver acquisition with volume acceleration
ICC: Inter-class correlation coefficient
CV: Coefficient of variation
SpA: Spondyloarthropathies
